# Preparing Europe for future health threats and crises ─ the European Medicines Agency; ensuring safe and effective medicines and medical devices

**DOI:** 10.2807/1560-7917.ES.2022.27.42.2200798

**Published:** 2022-10-20

**Authors:** Emer Cooke

**Affiliations:** 1 European Medicines Agency (EMA), Amsterdam, the Netherlands

**Keywords:** extended mandate, EMA, European Medicines Agency, preparedness, health crisis, medicine regulation, European Health Union

When coronavirus disease (COVID-19) spread rapidly across Europe in early 2020, the European Medicines Agency (EMA), together with the European Centre for Disease Prevention and Control (ECDC) and the European Commission, was at the forefront of the European Union (EU) response to the COVID-19 pandemic.

Despite the absence of an explicit legal mandate and appropriate resources, EMA quickly introduced a structure to coordinate activities and established processes which were crucial for an effective EU-wide response. For example, EMA coordinated the exchange of information and actions to identify and mitigate medicine shortages between EU countries when global pharmaceutical supply chains were disrupted almost overnight because of border closures and lockdowns and there was an increased demand for medicines, particularly in the intensive care unit setting. However, it became clear early in the pandemic that EMA needed stronger legal tools to help ensure the availability of medicinal products and medical devices and to support the development of new therapeutics to protect the health of Europeans in times of crisis.

EMA’s timely efforts and successes were subsequently recognised and codified in a new framework for a European Health Union put forward by the European Commission [1]. The framework includes three pillars: extending the mandate of both EMA and ECDC and establishing the European Commission’s new Directorate General HERA, the European Health Emergency Preparedness and Response Authority. Today, ECDC, EMA and HERA form part of a European preparedness matrix, in which each actor has its own role, while working closely together. This matrix aims to ensure a comprehensive approach to cooperation, coordination and communication across countries and EU bodies to protect EU citizens and address cross-border health threats.

The reinforced role of EMA in crisis preparedness and management is defined in Regulation (EU) 2022/123 [2]. The new responsibilities enable EMA to improve the availability of medicines and medical devices, whether it is dealing with shortages to ensure that already authorised medicines or devices remain available for EU patients in time of crisis, or whether it is supporting the development and authorisation of medicines and/or vaccines to address new public health emergencies [3].

Improving the availability of medicines authorised in the EU is a key priority for EMA and the European Medicines Regulatory Network, comprised of EMA, the EU countries’ national regulatory authorities and the European Commission.

Shortages of medicinal products became a major issue long before the COVID-19 pandemic. They often have complex root causes and have a serious impact on healthcare systems and infringe on the right of patients to access appropriate medical treatment. The extended mandate is an important step to address this growing threat to public health [2]. It builds on the work EMA started on a voluntary cooperative basis during the pandemic. While EMA had no formal role in managing these shortages, the agency set up an executive level steering group and engaged with single points of contact in each EU country and worked closely with companies, wholesalers and distributors to gain essential intelligence on the scope and underlying causes of the issues.

The extended mandate has provided EMA with the tools to officially deal with shortages. There is a new executive body, the Medicine Shortages and Safety Steering Group (MSSG), which comprises representatives from EMA and from countries, to respond robustly to medicine supply issues caused by major events or public health emergencies and to coordinate swift actions within the EU when needed [4] (Box).

Box **European Medicines Agency new mechanisms following the extension of its mandate on 25 January 2022**

- Medicine Shortages and Safety Steering Group (MSSG)
- Industry Single Point of Contact network (iSPOC)
- European shortages monitoring platform (ESMP)
- Data Analysis and Real-World Interrogation Network (DARWIN EU)
- Medicines Shortages Single Point of Contact (SPOC) Working Party
- Emergency Task Force (ETF)

Source [2].

In practice, the MSSG establishes lists of critical medicines needed during a major event or a public health emergency that require close monitoring of supply and demand with a view to identifying any actual or potential shortages of those medicinal products. EMA has already published lists of critical medicines for COVID-19 [5] and monkeypox [6]. These lists impose obligations on the marketing authorisation holders, who are required to update EMA for example on potential or actual shortages of the medicines listed.

The pharmaceutical industry plays an important role in the prevention and management of shortages, which is reflected in the updated and formalised Industry Single Point of Contact network (iSPOC). By 2 September 2022, every marketing authorisation holder in the EU had to register an iSPOC to enable rapid communication between EMA and companies [7]. Companies that market products included on specific lists of critical medicines now have new monitoring and reporting responsibilities in respect to forecasts of stock or demand. Regulation (EU) 2022/123 also foresees the development of a centralised electronic platform for pharmaceutical companies and countries to report on shortages, the European shortages monitoring platform (ESMP), by 2025. The ESMP is expected to induce better coordination, monitoring and prevention and could serve as the basis for extended EU-wide monitoring also in normal times [8].

The rapid spread of COVID-19 led to a sharp increase in demand for medical devices such as ventilators, surgical masks and COVID-19 test kits. However, the limited capacity to increase production and the vulnerability of the global supply chains caused severe supply disruptions and serious shortages. EMA’s new mandate aims to establish a solid and effective monitoring of medical devices shortages that can occur during a public health emergency [2].

Medical devices do not have a centralised authorisation procedure, but EMA is involved in the regulatory process [9]. The new mandate transfers the coordination of the medical device expert panels from the European Commission to EMA. This will lead to a more integrated approach, with one scientific agency managing both medicines and certain types of medical devices [10]. In the future, the panels’ opinions will be published in the dedicated European database on medical devices (EUDAMED) [11]. 

When confronted with a new disease, such as COVID-19, the ability to bring together all relevant actors and facilitate the processes of medicine development and regulation is key to accelerate the development and evaluation of new medicines. The need for integration across all regulatory fields is embodied in the Emergency Task Force (ETF), which pools the necessary scientific expertise across the EU [12]. The ETF provides scientific advice and reviews evidence on medicines that could be used for prevention or treatment during a public health emergency. It also offers scientific support to facilitate clinical trials, particularly large, well-designed multinational trials, and supports EMA’s scientific committees with the authorisation and safety monitoring of medicines and with recommendations on the use of medicines before authorisation.

The COVID-19 pandemic put a spotlight on the need to invest in and leverage real-world evidence to support crisis preparedness and response. The EMA new mandate provides a legal basis for the establishment of the Data Analysis and Real-World Interrogation Network (DARWIN EU), which will provide EMA’s scientific committees with real-world evidence from healthcare databases across the EU. EMA will be the principal user of DARWIN EU [13].

In the area of medical countermeasures, the scientific advice and regulatory support provided by EMA will be complemented by the financial support provided by HERA, which will leverage funding to support both basic, translational and clinical research and development susceptible to bring innovative medical countermeasures on the EU market.

The EU institutions have demonstrated considerable resilience in the face of COVID-19 [14], but more needs to be done. A large-scale emergency response requires a wide pool of expertise that can be drawn on at any time. The pandemic exposed the challenge of retaining sustainable human resources to answer to the increased and urgent demand for evaluation, as it is not feasible to rapidly recruit many new specialists in the specific area of infectious diseases while a crisis is ongoing. An agile network needs to be established to ensure that sufficient resources in terms of both staff and finances are available. Further, EMA will need to ensure planning and resourcing in new areas of expertise such as medical devices and diagnostics.

In a pandemic, nobody is safe before everyone is safe. EMA has to ensure that the needs and expectations of partners and stakeholders are carefully listened to. Acknowledging the uniqueness and complexity of the EU context, the agency will collaborate across the European medicines regulatory network and with public health authorities to strengthen EU-wide communications in a way that is transparent and strengthens trust.

The pandemic has highlighted the power of science and international cooperation. EMA has been working at high speed and in close cooperation with regulators, scientists and governments to respond effectively to the public health emergency. The European Health Union is based on the reinforced cooperation between EMA, ECDC and HERA.

Already during the pandemic, EMA has deepened its cooperation with ECDC, in particular in epidemiological forecasting, as this supports EMA’s regulatory processes. Further, regular communication at different levels feeds into the agencies’ strategic planning and decision-making and ensures aligned messaging during a health crisis. Together, EMA and ECDC are responsible for an initiative to strengthen post-marketing monitoring of the safety, effectiveness and impact of COVID-19 vaccines in the EU/European Economic Area [15]. The jointly coordinated, EU-wide effectiveness and safety studies are essential tools to monitor how novel vaccines perform in real life.

COVID-19 was the first public health emergency where countries agreed to pool their powers and budget and purchase medicines and vaccines in a joint and coordinated manner. The cooperation and information exchange between HERA and EMA is crucial for effective EU action.

The extended mandate makes EMA better equipped to deal with future emergencies. A practical example: EMA will work with ECDC to obtain epidemiological data to help forecast medicines needs and to request specific data from countries and supply-chain stakeholders [16]. The forecasts provide important input for HERA to build surge EU manufacturing capacities and stockpiles as well as launch emergency procurements and emergency deployment of medical countermeasures such as vaccines [17]. The ongoing monkeypox outbreak is the first instance in which all the new tools are applied.

Further, regular discussions between EMA and HERA collaborate within the Medicines Shortages Single Point of Contact (SPOC) Working Party, which is responsible for monitoring and reporting events that could affect the supply of medicines in the EU. In joint meetings, HERA regularly presents their surveys and studies on medicine availability and supply chain vulnerabilities, which provide important information from and for countries. Having HERA on board helps to implement these critical activities and has clarified the scope of the surveys.

COVID-19 will not be the last pandemic Europe will face [18]. In addition, there are already other threats to health, such as antimicrobial resistance and the effects of climate change. The cooperation and interaction between EMA, ECDC and HERA as envisaged by the EU Health Union should lead to better preparedness and more resilience to overcome new challenges together.

## Note

Emer Cooke is the Executive Director of the European Medicines Agency (EMA).

## References

[1] European Commission (EC). European Health Union. Brussels: EC. [Accessed: 19 Oct 2022]. Available from: https://ec.europa.eu/info/strategy/priorities-2019-2024/promoting-our-european-way-life/european-health-union_en

[2] Regulation (EU) 2022/123 of the European Parliament and of the Council of 25 January 2022 on a reinforced role for the European Medicines Agency in crisis preparedness and management for medicinal products and medical devices (Text with EEA relevance). Luxembourg: Official Journal of the European Union. 31 Jan 2022. Available from: https://eur-lex.europa.eu/legal-content/EN/TXT/?uri=CELEX%3A32022R0123

[3] European Medicines Agency (EMA). Regulation on EMA’s extended mandate becomes applicable. Amsterdam: EMA; 1 Mar 2022. Available from: https://www.ema.europa.eu/en/news/regulation-emas-extended-mandate-becomes-applicable

[4] European Medicines Agency (EMA). Executive Steering Group on Shortages and Safety of Medicinal Products (MSSG). Amsterdam: EMA; 11 May 2022. Available from: https://www.ema.europa.eu/en/about-us/what-we-do/crisis-preparedness-management/executive-steering-group-shortages-safety-medicinal-products

[5] European Medicines Agency (EMA). List of critical medicines for COVID-19 public health emergency (PHE) under Regulation (EU) 2022/123. Available from: https://www.ema.europa.eu/en/documents/other/list-critical-medicines-covid-19-public-health-emergency-phe-under-regulation-eu-2022/123_en.pdf

[6] European Medicines Agency (EMA). List of critical medicines for Monkeypox public health emergency (PHE) under Regulation (EU) 2022/123. Available from: https://www.ema.europa.eu/en/documents/other/list-critical-medicines-monkeypox-public-health-emergency-phe-under-regulation-eu-2022/123_en.pdf

[7] European Medicines Agency (EMA) Medicines Shortages Single Point of Contact (SPOC) Working Party . Available from: https://www.ema.europa.eu/en/committees/working-parties-other-groups/medicines-shortages-single-point-contact-spoc-working-party

[8] Ferreira J. Monitoring and mitigating shortages of medicines and devices. Presentation. Amsterdam: European Medicines Agency. [Accessed: 19 Oct 2022]. Available from: https://www.ema.europa.eu/en/documents/presentation/presentation-session-1-monitoring-mitigating-shortages-medicines-devices-j-ferreira-ema_en.pdf

[9] European Medicines Agency (EMA). Medical Devices. Amsterdam: EMA. [Accessed: 19 Oct 2022]. Available from: https://www.ema.europa.eu/en/human-regulatory/overview/medical-devices

[10] Dias SDR. Coordinating expert panels on high-risk medical devices and in vitro diagnostics. Amsterdam: European Medicines Agency (EMA). [Accessed: 19 Oct 2022]. Available from: https://www.ema.europa.eu/en/documents/presentation/presentation-session-3-coordinating-expert-panels-high-risk-medical-devices-vitro-diagnostics-s-da_en.pdf

[11] European Commission. EUDAMED - European Database on Medical Devices. Brussels: European Commission. [Accessed: 19 Oct 2022]. Available from: https://ec.europa.eu/tools/eudamed/#/screen/home

[12] European Medicines Agency (EMA). Emergency Task Force (ETF); Available from: https://www.ema.europa.eu/en/committees/working-parties-other-groups/emergency-task-force-etf

[13] European Medicines Agency (EMA). Data Analysis and Real World Interrogation Network (DARWIN EU). Amsterdam: EMA. [Accessed: 19 Oct 2022]. Available from: https://www.ema.europa.eu/en/about-us/how-we-work/big-data/data-analysis-real-world-interrogation-network-darwin-eu

[14] Peseckyte G. EU institutions showed ‘considerable resilience’ during pandemic, audit finds. Brussels: Euractiv; 1 Sep 2022. Available from: https://www.euractiv.com/section/health-consumers/news/eu-institutions-showed-considerable-resilience-during-pandemic-audit-finds/

[15] European Medicines Agency (EMA). EMA and ECDC join forces for enhanced post-marketing monitoring of COVID-19 vaccines in Europe. Amsterdam: EMA; 26 Apr 2021. Available from: https://www.ema.europa.eu/en/news/ema-ecdc-join-forces-enhanced-post-marketing-monitoring-covid-19-vaccines-europe

[16] Wathion N, Frias Z. How is EMA preparing for an extended mandate. Presentation at the PCWP/HCPWP Joint meeting. Amsterdam: EMA; 1 Jun 2021. Available from: https://www.ema.europa.eu/en/documents/presentation/presentation-how-ema-preparing-extended-mandate-nwathion-zfrias-ema_en.pdf

[17] European Commission. Questions and Answers: European Health Emergency preparedness and Response Authority – HERA. Brussels: European Commission 16 Sep 2021. Available from: https://ec.europa.eu/commission/presscorner/detail/en/qanda_21_4733

[18] European Commission. Horizon: The EU Research and Innovation Magazine. Q&A: Future pandemics are inevitable, but we can reduce the risk. Brussels: European Commission; 16 Dec 2021. Available from: https://ec.europa.eu/research-and-innovation/en/horizon-magazine/qa-future-pandemics-are-inevitable-we-can-reduce-risk

